# Gelation Performance of HPAM-Cr^3+^ Gels for Reservoir Profile Control: The Impact of Propagation Distance and Optimization Design

**DOI:** 10.3390/gels11110872

**Published:** 2025-10-31

**Authors:** Mengyun Li, Junjie Hu, Xiang Wang, Guicai Zhang

**Affiliations:** 1 College of Petroleum Engineering, China University of Petroleum, Qingdao 266580, China; 15689431951@163.com (M.L.); junjiehu2021@163.com (J.H.); wxiang9898@163.com (X.W.); 2 State Key Laboratory of Deep Oil and Gas, China University of Petroleum, Qingdao 266580, China

**Keywords:** HPAM-Cr^3+^ gels, propagation distance, gelation performance, over-crosslinking, gel dosage design

## Abstract

HPAM-Cr^3+^ (partially hydrolyzed polyacrylamide-chromium ion) gels are widely used in enhancing oil recovery (EOR) due to their advantages of low cost, controllability, and high strength. The propagation distance of gels within the reservoir significantly negatively impacts their gelation performance. However, the extent of this influence remains unclear, hindering precise optimization for field applications. This study first established a gelation performance characterization method based on visual inspection, rheological parameters, and long-term stability, accurately classifying gels into five types: stable strong gel (SSG), stable weak gel (SWG), colloidal dispersion gel (CDG), unstable gel (USG), and over-crosslinked gel (OCG). Subsequently, cross-experiments were conducted using varying concentrations of HPAM and Cr^3+^. Based on the contour map of visual appearance, storage modulus (G′), and water loss rate (R_w_) of the gels, distribution maps of gel morphology versus concentration were constructed. The gel performance was found to depend on the HPAM concentration and the crosslinking ratio (molar ratio of HPAM carboxyl groups to Cr^3+^ ions). No gel formation occurred when the HPAM concentration was below 800 mg/L, while concentrations above 2500 mg/L effectively inhibited over-crosslinking. The crosslinking ratio range for forming SSG was 5.56 to 18.68, with an optimal value of 9.27. Furthermore, the effect of propagation distance on gelation performance was investigated through 60 m sand-packed flow experiments. Results indicated that the minimum value of the crosslinking ratio was 2.632, the stable SSG formed when the propagation distance was less than 21 m, SWG formed within the 21–34 m range, and no intact gel formed beyond 34 m. It means that only the first 35% of the designed distance formed effective SSG for plugging. Finally, an optimization method for gel dosage design was established based on the findings. This method determines the optimal gel dosage for achieving effective plugging by calculating the volume of crosslinking system passing through the target fluid diversion interface and referencing the gel morphology distribution maps. These findings provide a straightforward and effective approach for the precise design of in-depth profile control agents.

## 1. Introduction

Water flooding is the dominant secondary recovery technology widely adopted worldwide. However, due to reservoir heterogeneity, injected water tends to channel through high-permeability pathways during water flooding development, resulting in the enrichment of substantial remaining oil in low-permeability zones that cannot be effectively swept. This leads to low displacement efficiency and failure to achieve the expected recovery factor [1,2,3,4]. To address this issue, profile control and water shutoff technologies capable of achieving deep fluid diversion in reservoirs have become a research focus for enhancing sweep efficiency and oil recovery [5]. Among various chemical profile control agents, gel systems formed by partially hydrolyzed polyacrylamide (HPAM) and chromium ion (Cr^3+^)-based crosslinkers have been extensively applied in major oil fields for decades, owing to their favorable comprehensive performance and relatively low cost [6,7].

Compared to other common profile control and water shutoff systems, the HPAM/Cr^3+^ system possesses distinct advantages. Firstly, the HPAM/Cr^3+^ system is relatively inexpensive. Its application cost is more suitable for the large-volume injections required in mature oil fields compared to many modern polymer systems or nanomaterials [8,9,10]. Secondly, the gelation process of the HPAM/Cr^3+^ system is controllable. By adjusting parameters such as polymer and crosslinker concentration, pH, and temperature, the gelation time of the system can be regulated to meet specific field requirements. This ensures the gelant can reach deep reservoir regions before setting, preventing its accumulation and gelation near the injection wellbore [11,12,13]. Furthermore, the gel formed by the HPAM/Cr^3+^ system exhibits considerable viscoelasticity and mechanical strength, enabling it to effectively block high-permeability pathways and divert subsequent flooding fluids [14,15,16].

Despite the widespread application and evident advantages of the HPAM/Cr^3+^ system, its in-depth penetration efficiency and deep plugging strength during field operations often fall short of expectations [17,18]. Many researchers contend that the actual gelation capability of the HPAM/Cr^3+^ system in the deep reservoir is questionable. The gelation reaction is highly sensitive to the reservoir environment. Multiple factors, including temperature, salinity, formation water hardness, and the shear experienced by the gelant during propagation, can significantly impact the system’s ultimate plugging effectiveness [19,20,21]. Cr^3+^ ions may be prematurely consumed through hydrolysis, precipitation, or adsorption onto the surface of formation rocks. This prevents them from effectively reaching the target zone, resulting in a substantially reduced crosslinker concentration by the time the solution arrives in the deep reservoir [22,23,24,25,26]. Consequently, the system may form an unstable gel or fail to gel entirely at depth, leading to the failure of the profile control treatment. In recent years, numerous studies have addressed these challenges. For instance, Zhao (2021) conducted model experiments to estimate the maximum propagation distance of gel in channels [26]. His work provided a comprehensive assessment of gel material effectiveness and enabled quantitative calculations of gel retention within porous flow channels. Similarly, Liang et al. (2022) [27] demonstrated, through long-distance migration experiments in a 20 m sand-pack tube, that gel strength became very low and gelation was difficult when the migration distance exceeded 8 m. This highlights a common issue in achieving deep diversion using crosslinked systems in many reservoirs [28].

Given the uncertainty associated with the in-depth gelation of the HPAM/Cr^3+^ system, merely testing parameters such as solution concentration and gel strength under laboratory conditions is insufficient. To ensure the effectiveness of deep fluid diversion, it is essential to experimentally validate its gelation behavior and plugging performance under conditions that simulate migration and placement within the target reservoir zone. Beyond gelation tests under static conditions, it is more critical to evaluate in-depth gelation and plugging through gelant propagation and displacement experiments [29,30,31,32,33]. While most existing studies have qualitatively or semi-quantitatively revealed the phenomenon of “performance degradation due to propagation,” they have failed to systematically establish the quantitative relationship between “propagation distance” and the “final gel morphology and strength.” Specific data and conclusions regarding the distance at which a stable strong gel degrades into a weak gel, or the critical distance beyond which gelation fails entirely, remain scarce. This knowledge gap hinders the precise optimization of gel dosage and injection parameters in field applications.

In parallel with polymer/gel-based profile control, there is rapidly growing interest in nanofluid-based conformance control and EOR formulations. Recent studies have shown that nanoparticle-stabilized injection fluids can be utilized to regulate thermophysical properties, alter reservoir wettability, and modify interfacial tension in porous media. This leads to effective conformance control and enhanced sweep efficiency. In a SiO_2_–water nanofluid system, simultaneous optimization of nanoparticle volume fraction, injection temperature, and flow rate in a heterogeneous porous medium produced up to 27% higher oil recovery compared with conventional water flooding, possessing significant potential for widespread application. However, the mechanisms of conventional nanoparticle-fluid systems have primarily focused on enhancing sweep efficiency by modifying flow characteristics and interfacial mechanics; they typically do not form mechanically stable blocking structure tens of meters away from the wellbore [34]. Beyond this, other in-depth profile control technologies—such as preformed particle gels (PPG), colloidal dispersion gels (CDG), BrightWater-type, and thermosensitive polymer systems—are also capable of forming a blocking effect deep within the reservoir, thereby enhancing ultimate recovery [35,36,37].

By contrast, the present work explicitly targets deep mechanical conformance control using HPAM–Cr^3+^ gels. This study aims to quantitatively investigate the evolution of gelation performance in HPAM/Cr^3+^ systems with propagation distance through systematic experimentation, addressing the research gap in quantitative design methodology. By conducting propagation experiments of the HPAM/Cr^3+^ crosslinking system in sand-pack models, we observed its dynamic transport behavior, measured system parameters at locations representing different reservoir positions, and analyzed how propagation distance and other factors influence final gelation performance and plugging effectiveness. Furthermore, we determined the required injection concentration and volume to ensure effective plugging efficiency under the gel state conditions necessary for in-depth fluid diversion. This research ultimately seeks to develop an optimized design method for gel dosage by precisely quantifying the critical transition points in gel morphology and strength at different propagation distances. The findings provide enhanced theoretical foundation and comprehensive experimental data to support the application of HPAM/Cr^3+^ systems for in-depth conformance control, offering practical significance for enhancing recovery in mature, high-water-cut oilfields.

## 2. Results and Discussion

This study has made research progress in the following three aspects through experiments and theoretical analysis.

(1) Definition and Classification of Gel System

It is clear that HPAM-Cr^3+^ cross-linking system can form five gel states with significant differences, including stable strong gel (SSG), stable weak gel (SWG), colloidal dispersed gel (CDG), unstable gel (USG) and over cross-linked gel (OCG). This classification is based on multidimensional parameters such as elastic modulus (G′) and fluid loss rate (R_w_) and visual appearance, breaking through the traditional common judgment method based solely on apparent morphology. From the perspective of field application: SSG type gel can form a continuous blocking barrier, which is suitable for deep profile control and displacement; SWG and CDG exhibit weaker plugging capacity, while OCG is characterized by high brittleness, higher water loss rate, and a tendency for premature gelation near the wellbore.

(2) Quantitative Relationship Between Injection Concentration and Placement Depth

By correlating gel state with polymer concentration and crosslinking ratio, and through 60 m sand-pack flooding experiments, critical thresholds were identified for gel-state transitions during long-distance propagation: SSG forms within approximately 21 m; weak or partially continuous gels are observed between 21–34 m; and beyond 34 m, no continuous plugging structure develops. This phenomenon of attenuation with distance is due to the dilution/retention effect during migration and polymer degradation caused by shear stress, resulting in a synchronous decrease in the effective concentration and cross-linking ability of the cross-linking system in the deep reservoir zones.

(3) Design Methodology and Field Application Value

Based on experimental results, a design guideline for over-injection was established to pre-determine the required concentration and dosage for effective deep plugging. This approach ensures effective gel placement in the target depth, prevents excessive gelation near the wellbore, and maximizes economic benefit by balancing chemical cost against anticipated incremental oil recovery.

In summary, this work bridges the gap between laboratory observations and field-implementable quantitative design, providing a theoretical foundation and a practical optimization tool for in-depth profile control and displacement operations.

### 2.1. Characterization of the HPAM-Cr^3+^ Crosslinking Reaction Product

#### 2.1.1. Reaction Mechanism of HPAM-Cr^3+^ Crosslinking

**Figure 1:**
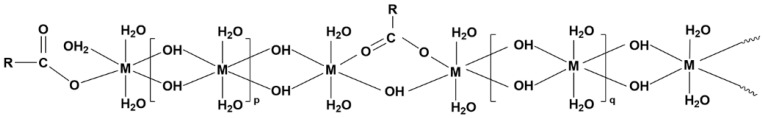
Schematic Illustration of the HPAM-Cr^3+^ Crosslinking Reaction Mechanism.

Figure 1 illustrates the schematic diagram of the HPAM-Cr^3+^ crosslinking reaction mechanism. Chromium ions undergo hydration to form polynuclear hydroxyl-bridged complex ions, which subsequently crosslink with the carboxyl groups of polymer molecules to form a gel. The crosslinking reaction of the chromium gel is a two-stage process, consisting of a fast reaction stage followed by a slow reaction stage [38,39,40].

Based on the aforementioned crosslinking reaction, this study defines the crosslinking ratio as the molar ratio of HPAM carboxyl group segments to Cr^3+^ ions in the HPAM-Cr^3+^ system. The minimum crosslinking ratio is defined by the formation of a hydroxyl bridge through the dehydration between two carboxyl groups from HPAM and one carboxylated Cr^3+^ ion. The calculation formula is shown in Equation (1).

(1) $$B=0.2138\times \frac{w_{HPAM}}{w_{{Cr}^{3+}}}$$

#### 2.1.2. Characterization of HPAM-Cr^3+^ Crosslinked Gel Properties

**Figure 2:**
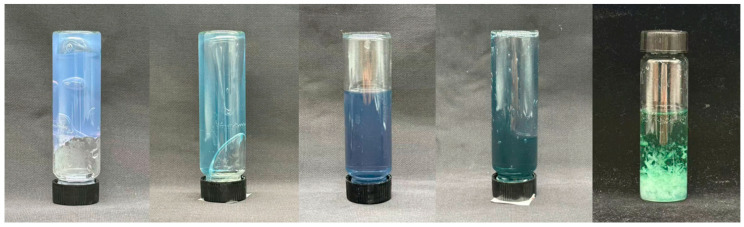
Schematic Illustration of Gel Morphologies for Different Performance Categories.

Previous studies have revealed that the properties of HPAM-Cr^3+^ crosslinking reaction products can be categorized into five distinct types based on their structural integrity and stability: Stable Strong Gel (SSG), Stable Weak Gel (SWG), Colloidal Dispersion Gel (CDG), Unstable Gel (USG), and Over-crosslinked Gel (OCG). Their visual appearances are illustrated in Figure 2.

- SSG: This type possesses a moderate crosslinking density, forming a robust three-dimensional network structure. It exhibits excellent long-term stability, maintaining its integrity and water-holding capacity over extended periods.
- SWG: Characterized by a low crosslinking density, SWG forms a weak three-dimensional network. It demonstrates good long-term stability but has a relatively poor water-binding capacity due to its less cohesive structure.
- CDG: CDG exhibits low viscosity and consists of weakly crosslinked HPAM microgels or a small number of HPAM molecules forming gel-like particles without a continuous, bulk three-dimensional network. Despite the lack of a macroscopic network, it shows good long-term stability.
- USG: USG typically appears as a turbid, viscoelastic mass or a coexisting system of water and gel phases resulting from local syneresis or breakdown. It suffers from poor long-term stability, attributed to the continuous shrinkage of the initially formed network over time. This process expels bound water, reduces the effective gel volume, and may eventually lead to complete gel disappearance.
- OCG: Paradoxically, OCG has a viscosity lower than that of an HPAM solution at the same concentration. OCG refers to a formulation system where the HPAM carboxylate-Cr^3+^ molar ratio falls significantly below the optimal crosslinking window. Under such non-ideal conditions, excess Cr^3+^ triggers a sharp increase in local crosslinking density, forming highly concentrated ultra-dense coordination nodes. This imbalanced crosslinking mechanism causes spatial collapse of polymer chains, ultimately resulting in the formation of rigid, over-tightened isolated aggregates rather than a continuous and stable three-dimensional network. This structural degradation process severely compromises the gel’s macroscopic plugging capacity and long-term stability, it decreases both bound water and solvation water within the system, leading to pronounced syneresis and high static fluid-loss rate [41].

Currently, no unified methodology exists for characterizing the performance of HPAM-Cr^3+^ crosslinking reaction products. The visual strength code method is operationally simple but fails to quantitatively represent gel strength. Viscosity-based methods (capillary viscometry and rheometric viscosity measurement) are only suitable for assessing relatively weak gel strengths and cannot determine long-term stability. Pressure differential characterization methods (transition pressure difference, breakthrough pressure difference, breakthrough vacuum degree) lack standardized protocols, resulting in poor data comparability, and also cannot ascertain long-term stability. The rheological parameter method, which utilizes the storage modulus to evaluate gel strength, is applicable for measuring a wide range of gel strengths but similarly cannot determine long-term stability. This study integrates the rheological parameter method, visual method, and gel long-term stability to establish a comprehensive characterization methodology for gel performance. Table 1 presents the corresponding parameters for gels of different performance categories.

- SSG: Appears as a homogeneous, transparent viscoelastic solid (Appearance Code 1), with an elastic modulus (G′_gel_) ≥ 10 Pa and a fluid loss rate (R_w_) ≤ 15%.
- SWG: Appears as a homogeneous, transparent viscoelastic solid (Appearance Code 1), with G′_gel_ ≥ G′_HPAM_(the storage modulus of an HPAM solution at the same concentration as the gel system), G′_gel_ < 10 Pa, and R_w_ ≥ 15% (where R_w_ is greater than or equal to the fluid loss rate of the HPAM solution at the same concentration).
- CDG: Appears as a homogeneous, transparent fluid (Appearance Code 1), with G′_gel_ < G′_HPAM_ and R_w_ ≥ $R_{w}^{HPAM}$.
- USG: Appears as a turbid viscoelastic solid (Appearance Code 1–2), with G′_gel_ ≥ G′_HPAM_ and R_w_ ≥ 15%.
- OCG: Appears as a heterogeneous fluid (Appearance Code 3), with G′_gel_ < G′_HPAM_ and R_w_ ≥ $R_{w}^{HPAM}$ [42,43].

### 2.2. Diagnostic Chart for HPAM-Cr^3+^ Crosslinked Reaction Products

**Figure 3:**
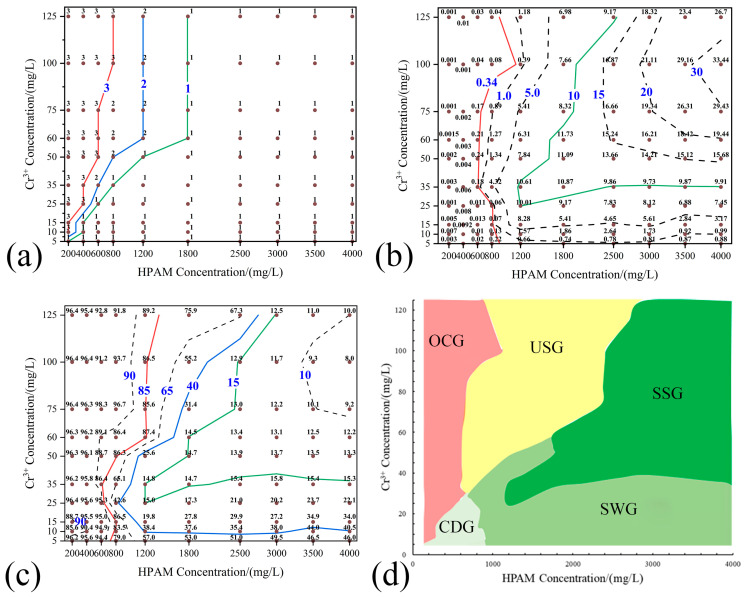
Contour Maps of Gel Properties and Morphology Distribution under Different HPAM and Cr^3+^ Concentrations. (**a**) Visual Appearance Code (1: Homogeneous & Transparent. 2: Turbid. 3: Flocculent). (**b**) G′, Pa; (**c**) R_w_, %. (**d**) Distribution Map of Crosslinked Product Morphologies.

A cross-experiment involving 100 combinations of HPAM concentrations (200–4000 mg/L) and Cr^3+^ concentrations (5–125 mg/L) was conducted, resulting in a crosslinking ratio (molar ratio of HPAM carboxyl groups to Cr^3+^) ranging from 0.37 to 185.4. The FGT of the HPAM-Cr^3+^ systems was determined at 60 °C. Figure 3a–c present contour maps of the visual appearance, G′, and R_w_ at FGT, respectively. By integrating these contour maps using the previously established performance characterization method for HPAM-Cr^3+^ reaction products, a distribution map of the five distinct morphologies was generated, as shown in Figure 3d. This map provides a crucial basis for selecting appropriate formulations for the HPAM-Cr^3+^ gel system in practical applications. In the severe over-crosslinking (OCG) region, the system forms localized, brittle, high-modulus aggregates rather than a continuous viscoelastic gel barrier, accompanied by high fluid loss. This formulation tends to undergo premature gelation near the wellbore, consuming significant amounts of polymer and crosslinker while increasing the risk of near-wellbore plugging. Consequently, it fails to establish an effective barrier in the deep reservoir, making it operationally unfavorable.

Figure 3d should be interpreted as a two-parameter diagnostic chart rather than as a set of isolated, non-overlapping “recipes.” Although HPAM concentration significantly influences gel development, the final gel state is jointly determined by both HPAM concentration and crosslinking ratio, and is further modified by dilution/retention effects and shear during propagation. Consequently, different gel states partially overlap in HPAM concentration ranges. At similar HPAM concentrations, improper crosslinking ratios or severe propagation-induced degradation may lead to the formation of SWG/USG instead of SSG. Due to this overlapping nature, assigning a single “HPAM concentration range” to each gel state would be misleading. The correct application of Figure 3d involves: (1) identifying HPAM concentration and crosslinking ratio combinations that fall within the target gel region, and (2) referring to the corresponding summary table of visual characteristics, storage modulus (G′), and fluid-loss rate (R_w_) to evaluate the mechanical integrity and sealing potential of the gel.

### 2.3. Factors Influencing the Performance of HPAM-Cr^3+^ Crosslinking Reaction Products

#### 2.3.1. Effects of HPAM Concentration

**Figure 4:**
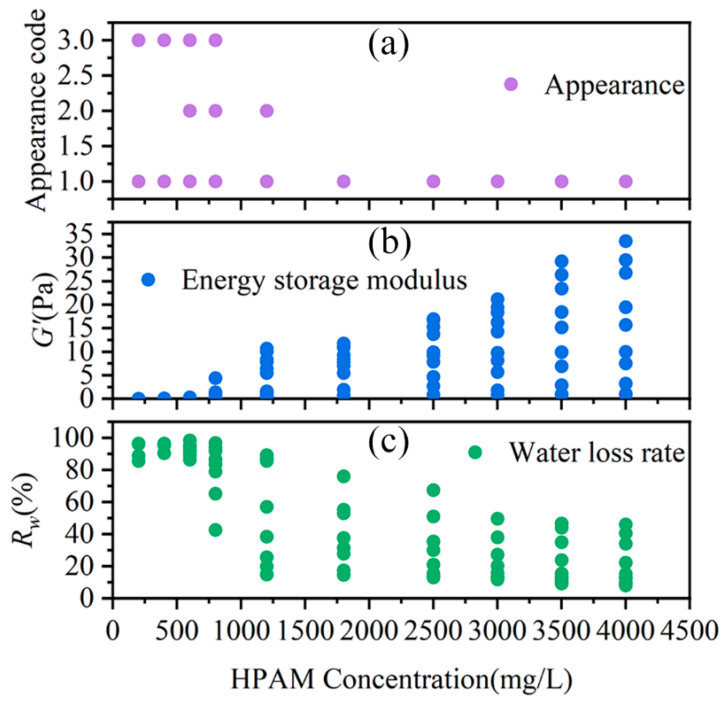
Effect of HPAM Concentration on the Performance of the HPAM-Cr^3+^ Crosslinking System. (**a**) Visual appearance of the crosslinked systems; (**b**) G′ of the crosslinked systems; (**c**) R_w_ of the crosslinked systems.

Figure 4a–c depict the variations in the appearance, G′, and R_w_ of the HPAM-Cr^3+^ crosslinking system with respect to HPAM concentration, respectively. Figure 4a,c show that as HPAM concentration increases, the visual strength of the crosslinked system increases, and the water loss rate decreases. This indicates that systems with low HPAM concentrations (<800 mg/L) fail to form a stable gel, while high HPAM concentrations (>2500 mg/L) effectively suppress severe over-crosslinking. This is attributed to the reduced number of polymer chains bearing stress and crosslinking sites at lower polymer concentrations, preventing the formation of a stable, continuous three-dimensional network structure. Figure 4b shows that the G′ of the crosslinked system exhibits an increasing trend with rising HPAM concentration. The combined data from Figure 4b,c demonstrate that increasing the HPAM concentration leads to enhanced gel strength and improved stability. This is due to the dual enhancement of the density of gel network structural units (chains, crosslinking points) and the physical interactions resulting from the increased polymer concentration, which collectively strengthen the network’s elastic response capability and the system’s ability to immobilize water within its structure.

#### 2.3.2. Effects of Crosslinking Ratio

**Figure 5:**
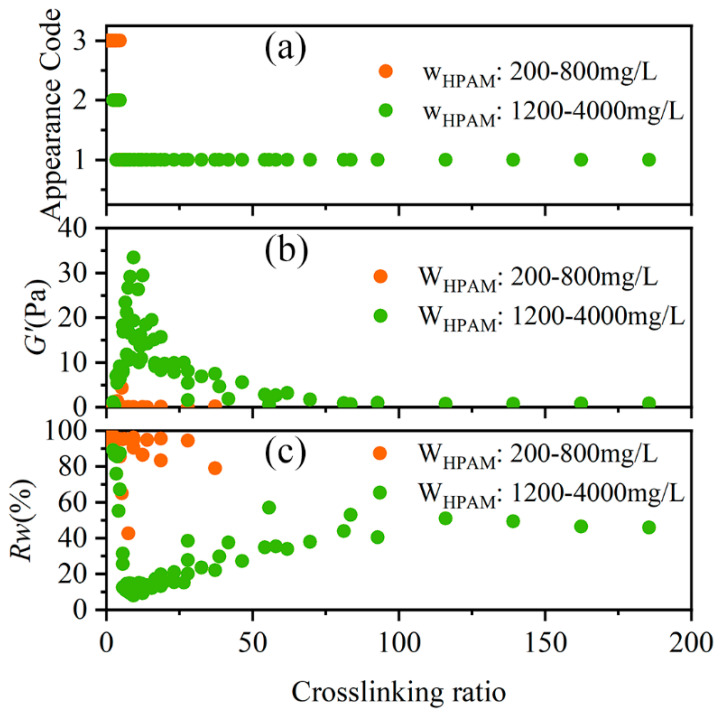
Effect of Crosslinking Ratio on the Performance of the HPAM-Cr^3+^ Crosslinking System. (**a**) Visual appearance of the crosslinked systems; (**b**) G′ of the crosslinked systems; (**c**) R_w_ of the crosslinked systems.

Figure 5a illustrates the effect of the crosslinking ratio on the visual appearance of the crosslinked systems. Severe over-crosslinking was observed in all systems when the crosslinking ratio was less than 2.23. In contrast, when the crosslinking ratio exceeded 4.64, the systems formed a homogeneous and transparent gel, indicating the suppression of severe over-crosslinking, regardless of the polymer concentration. Figure 5b,c describe the influence of the crosslinking ratio on G′ and water loss rate of the crosslinked systems, respectively. As the crosslinking ratio increased, both the storage modulus and the water loss rate exhibited a peak, suggesting the existence of an optimal crosslinking ratio for the HPAM-Cr^3+^ reaction. The crosslinking ratio range for forming an SSG was determined to be between 5.56 and 18.68, with an optimal value of 9.27. It is noteworthy that no stable strong gel formed when the HPAM concentration was below 800 mg/L.

### 2.4. Effect of Propagation Distance on Gelation Performance

**Figure 6:**
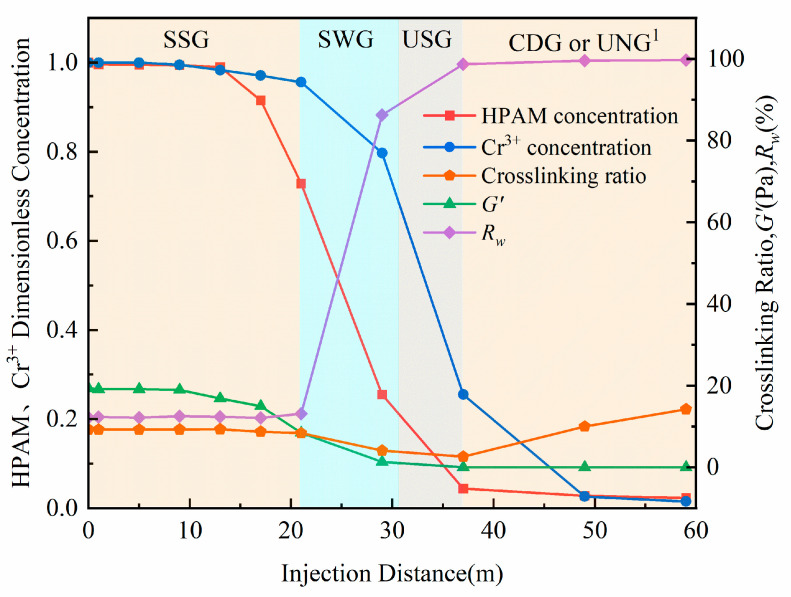
Gelation Performance at Different Propagation Distances in the Sand-Pack Model. 1—Ungelled.

To evaluate the impact of porous media propagation on the gelation performance of the HPAM-Cr^3+^ crosslinking system, a gelant solution was prepared using 3000 mg/L HPAM and 75 mg/L Cr^3+^, a composition selected from the SSG region of the performance map. Subsequently, 1 PV of the gelant was injected into a series of connected sand-packed tubes (total length: 60 m), and samples were collected at different locations along the tubes. Figure 6 presents the dimensionless concentrations of HPAM (sample concentration divided by initial concentration) and Cr^3+^, the crosslinking ratio, the G′ after gelation at 60 °C, and the R_w_ after gelation at 60 °C for these samples. As the crosslinking system propagated through the sand pack, the concentrations of HPAM and Cr^3+^ exhibited three distinct stages: a slow decline, followed by a rapid decrease, and then another period of slow decline. Notably, the HPAM concentration decreased more rapidly than that of Cr^3+^. At the outlet of the sand-pack system (the propagation front), the concentrations of both HPAM and Cr^3+^ dropped to nearly zero. This behavior is attributed to the mechanisms of adsorption, retention, and dilution occurring during their transport through the porous media. Meanwhile, the concentration reduction is also closely related to mechanical shear effects. During the injection of the crosslinking system and its flow through porous media, the high-molecular-weight HPAM chains experience intense shear and elongational stresses, leading to partial chain scission and disentanglement. This significantly reduces the apparent molecular weight of the polymer and shortens the length of carboxylate segments available for coordinating with Cr^3+^. Consequently, the gelant reaching deeper zones exhibits not only a lower effective concentration but also diminished crosslinking capability, manifesting as delayed gelation onset, reduced final G′, and a tendency to form SWG or CDG rather than SSG.

The differing rates of concentration decline for HPAM and Cr^3+^ led to a specific trend in the crosslinking ratio, which first decreased and then increased as propagation continued, reaching a minimum value of 2.632. This minimum value indicates that the system temporarily entered an unstable (over-crosslinked) region due to the shifting crosslinking ratio. The gelation performance varied significantly with propagation distance: SSG formed within 0–21 m; SWG formed between 21–34 m; and beyond 34 m, no intact gel structure formed. This performance distribution is a direct consequence of the changes in HPAM concentration, Cr^3+^ concentration, and the resulting crosslinking ratio.

In field applications for profile control and water shutoff, the gelant volume is typically designed based on the intended plugging distance. The results from this experiment indicate that for an injection of gelant designed to propagate 60 m, only the first 35% of the designed distance (0–21 m) formed effective SSG for plugging. A further 21.7% of the distance (21–34 m) formed less effective SWG, and a significant portion, 43.3% of the designed distance (beyond 34 m), failed to form any coherent gel, offering virtually no plugging effect in high-permeability channels. This clearly demonstrates that an over-design approach (injecting more than the theoretically required volume) is essential for the successful design of gel treatment volumes [44].

The objective and significance of this study extend beyond simply injecting polymer into the deep reservoir; they focus on forming an effective blocking body at the target depth to seal high-permeability channels. Only SSG exhibits the necessary high G′, low R_w_, and structural continuity to achieve optimal profile control and displacement performance. Neither SWG/CDG nor OCG can provide equivalent deep blocking or fluid diversion effects, thus offering limited contribution to sweep efficiency improvement. This is the fundamental reason why the research content of this article always revolves around the core issue of whether the target position can form SSG.

### 2.5. Study on Gel Dosage Design Methodology

In field design of conformance control treatments, the dosage and concentration of gelants are typically calculated based on the thickness of the extreme water channeling zones. Since the viscosity of the gelant solution increases with HPAM concentration, its initial injectability decreases accordingly. However, when the concentrations of HPAM and Cr^3+^ in the crosslinking system are low, the gel strength diminishes with increasing propagation distance. To compensate for this concentration loss, field practice often involves increasing the total gelant volume. Therefore, determining the required throughput of the crosslinking system to achieve ideal plugging performance within the target zone is critically important.

Based on the previously measured concentrations of HPAM and Cr^3+^ in the crosslinking system at different cross-sections along the sand-packed tube, and assuming a piston-like displacement of the system within the tube, the total injection volume ($Q_{c}$) and the cumulative propagation distance ($L=\frac{Q_{c}}{\varphi \pi r^{2}}$) are defined. (Here, *φ* represents porosity and *r* denotes the radius of the cross-section).

The throughput of the crosslinking system can be defined as $Q_{c}^{i}=Q_{c}\times (1-\frac{X_{i}}{L})=Q_{c}-0.248X_{i}$, where $X_{i}$ is the distance from a given cross-section to the injection point. During its propagation through the sand pack, the HPAM and Cr^3+^ in the crosslinking system are subject to adsorption, entrapment, and dilution. Under the combined influence of these mechanisms, the concentrations of HPAM and Cr^3+^ decrease with increasing propagation distance. Furthermore, previous studies have indicated that the retention of HPAM and Cr^3+^ in porous media is governed by their concentrations, retention time, sand grain composition, environmental conditions, etc. Therefore, under fixed experimental conditions, the retention amount at any given cross-section of the sand pack is positively correlated with the throughput of the crosslinking system.

#### 2.5.1. Optimization Method for HPAM Concentration

**Figure 7:**
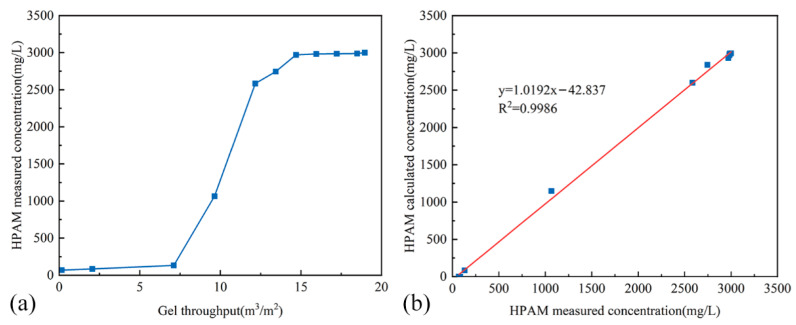
Diagrams of HPAM Concentration Data. (**a**) Measured HPAM concentration at the fluid diversion interface versus the throughput of the crosslinking system; (**b**) Correlation between calculated and measured HPAM concentrations.

Figure 7a illustrates the functional relationship between the throughput of the crosslinking system at the fluid diversion interface and the measured HPAM concentration at that interface. The HPAM concentration at a distance of *X_i_* meters from the injection point can be calculated using Equation (2), where $w_{HPAM}$ represents the initial concentration of HPAM in the crosslinking system prior to injection.

(2) $$w_{HPAM}=\frac{w_{HPAM}^{max}}{1+a{\left(Q_{c}^{i}\right)}^{b}}$$

Taking the logarithm of both sides of Equation (2) yields Equation (3). Linear regression of $log(\frac{w_{HPAM}^{max}-w_{HPAM}}{w_{HPAM}})$—$log(Q_{c}^{i})$ data was performed, yielding the calculated values of *a* = 3.628 × 10^7^ and *b* = −8.123.

(3) $$\log\left(\frac{w_{HPAM}^{max}-w_{HPAM}}{w_{HPAM}}\right)=b\log\left(Q_{c}^{i}\right)+\log a$$

Based on the above equation, the HPAM concentrations at various distances from the injection point were calculated. A plot of the calculated HPAM concentrations versus the measured concentrations is presented in Figure 7b. The results demonstrate a strong linear relationship between the calculated and measured values, with a slope of 1.0192 and a correlation coefficient of 0.9986, confirming the accuracy of the calculation method. Consequently, for field design of HPAM dosage, this method can be employed first to calculate the throughput and concentration of the crosslinking system. Subsequently, the gelation performance can be determined by consulting the performance chart (e.g., Figure 3d) to identify the required HPAM concentration in the gel for achieving optimal plugging performance at the target location.

#### 2.5.2. Optimization Method for Cr^3+^ Concentration

**Figure 8:**
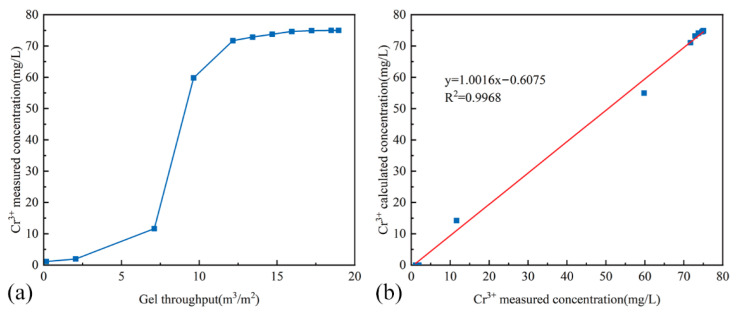
Diagrams of Cr^3+^ Concentration Data. (**a**) Measured Cr^3+^ concentration at the fluid diversion interface versus the throughput of the crosslinking system; (**b**) Correlation between calculated and measured Cr^3+^ concentrations.

Figure 8a presents the functional relationship between the throughput of the crosslinking system at the fluid diversion interface and the measured Cr^3+^ concentration at that interface. The Cr^3+^ concentration at a distance of *X_i_* meters from the injection point can be calculated using Equation (4), where $w_{Cr}^{max}$ represents the initial concentration of Cr^3+^ in the crosslinking system prior to injection.

(4) $$w_{Cr}=\frac{w_{Cr}^{max}}{1+a{\left(Q_{c}^{i}\right)}^{b}}$$

Taking the logarithm of both sides of Equation (4) yields Equation (5). Linear regression of $log(\frac{w_{Cr}^{max}-w_{Cr}}{w_{Cr}})$—$log(Q_{c}^{i})$ data was performed, yielding the calculated values of *a* = 1.349 × 10^10^ and *b* = 10.085.

(5) $$\log\left(\frac{w_{Cr}^{max}-w_{Cr}}{w_{Cr}}\right)=b\log\left(Q_{c}^{i}\right)+\log a$$

Based on the aforementioned equation, the Cr^3+^ concentrations at various distances from the injection point were calculated. The correlation between the calculated and measured Cr^3+^ concentrations is presented in Figure 8b. The results indicate a strong linear relationship between the calculated and measured values, with a slope of 1.0016 and a correlation coefficient of 0.9968, confirming the accuracy of the calculation method. Therefore, for the field design of Cr^3+^ dosage, this method can be employed to first determine the throughput and concentration of the crosslinking system. The corresponding gelation performance can then be identified by referring to the performance chart provided in Figure 3d, thereby determining the required Cr^3+^ concentration in the gel to achieve optimal plugging performance at the target location.

In field practice, the outcomes of this study enable operators to directly use the diagnostic chart to predict the gel state likely to form at a target depth and determine the required gel system concentration and volume to achieve SSG at that location. This approach allows for proactive design rather than relying solely on post-treatment production data for effectiveness evaluation.

To ensure the formation of an effective SSG-type gel at the target reservoir depth, the over-design approach—injecting a higher concentration crosslinking system—does indeed significantly increase economic costs. However, achieving effective in-depth profile control and displacement can enhance sweep efficiency and improve ultimate recovery, thereby generating economic returns. The design methodology proposed in this study establishes correlations between gel state, polymer concentration, and crosslinking ratio, links these gel states to propagation distance, and determines the minimum required injection concentration to form SSG at the specified depth. This enables precise guidance on chemical dosage for in-depth fluid diversion in field applications, preventing premature gelation near the wellbore and optimizing development outcomes while maintaining cost control.

## 3. Conclusions

1. A comprehensive gel characterization methodology was established by integrating visual inspection, rheological parameters, and long-term stability, enabling accurate classification of gels into five distinct categories: SSG, SMG, CDG, UMG, and OWG. This methodology was applied to combine contour maps of visual appearance, storage modulus (G′), and water loss rate, generating a morphology distribution map for HPAM-Cr^3+^ crosslinking reaction products. This map provides a critical basis for selecting appropriate formulations for the HPAM-Cr^3+^ gel system.
2. The factors influencing the performance of HPAM-Cr^3+^ crosslinking reaction products were systematically investigated, identifying HPAM concentration, crosslinking ratio, and system propagation distance as key parameters. Results demonstrated that no gel formation occurs at HPAM concentrations below 800 mg/L, while concentrations above 2500 mg/L effectively inhibit over-crosslinking. The crosslinking ratio range for forming SSG was determined to be 5.56 to 18.68, with an optimal value of 9.27. Sand-pack flow experiments over 60 m revealed that stable SSG forms within 21 m of propagation, SMG forms between 21–34 m, and no coherent gel forms beyond 34 m. This indicates that only the first 35% of the designed treatment distance develops effective SSG for plugging.
3. An optimized design method for gel dosage was developed based on this research. The method determines the optimal gel volume by calculating the crosslinking system throughput at the target fluid diversion interface and referencing the gel morphology distribution map. Furthermore, calculation formulas were provided to determine the required initial concentrations of HPAM and Cr^3+^ in the crosslinking system to achieve optimal plugging performance at the target location. This provides a straightforward and effective approach for the precise design of in-depth conformance control agents in oil reservoirs.
4. In light of the distance thresholds identified of this study, future studies should prioritize gel chemistries with enhanced shear resistance and reduced rock adsorption so that the concentration–ratio state remains within the SSG domain along extended flow paths. A second direction is time-programmed or protected crosslinkers to delay gelation until arrival while enabling rapid post-arrival strength build-up. Finally, embedding shear- and adsorption-corrections into our diagnostic chart via a coupled transport–rheology–adsorption model will allow prediction of an effective propagation distance under realistic velocity profiles and guide field-scale optimization.

## 4. Materials and Methods

### 4.1. Materials

**Figure 9:**
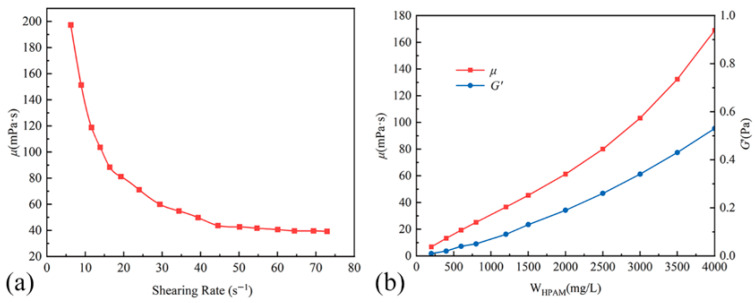
Variations in Viscosity and Storage Modulus of HPAM Solutions. (**a**) Viscosity of the HPAM solution as a function of shear rate. (**b**) Viscosity and Storage Modulus of HPAM solutions as a function of concentration.

Potassium dichromate solution (0.1 mol/L), sodium nitrite (AR, ≥99%), lactic acid (≥90%), propionic acid (AR, ≥99.5%), acetic acid (High Purity, ≥99.8%), and soluble starch were purchased from Shanghai Aladdin Biochemical Technology Co., Ltd (Shanghai, China). HPAM (with a reported weight-average molecular weight of 1.98 × 10^7^ g/mol, a hydrolysis degree of 26.31%, and a solid content of 93.80%) was supplied by Anhui Tianrun Chemical Industry Co., Ltd (Bengbu, China). Figure 9a illustrates the viscosity of the HPAM solution (3000 mg/L) as a function of shear rate at 60 °C. Figure 9b shows the viscosity (*μ*) and storage modulus (G′) of HPAM solutions versus concentration at 60 °C. The viscosity measurements were conducted at a fixed shear rate of 13.88 s^−1^, while the storage modulus was determined at an oscillatory frequency of 0.1 Hz and a stress of 0.2 Pa. Sodium formate (AR, 99.5%) and cadmium iodide (AR, 99.5%) were obtained from Macklin (Shanghai, China). Saturated bromine water was purchased from Beijing Huakesheng Fine Chemical Products Trade Co., Ltd. (Beijing, China). Synthetic formation water was prepared by dissolving salts in deionized water to match the ionic composition and concentration of the target oilfield’s brine.

### 4.2. Preparation of the Organic Chromium Crosslinker

The organic chromium crosslinker (designated as OCR-1) was prepared as follows. Lactic acid, propionic acid, and acetic acid were sequentially added to a temperature-controlled reactor equipped with a condenser. Potassium dichromate was then introduced into the reactor and allowed to dissolve completely under constant stirring. The reaction system was subsequently heated to a temperature range of 70–90 °C, after which sodium nitrite was added slowly and in batches. The reaction was maintained at this temperature for 8–12 h. Upon completion, the mixture was cooled to room temperature, yielding the final product, OCR-1, as a dark green, viscous solution. The resulting crosslinker had a Cr^3+^ mass fraction of 4.89% and a molality of 0.94 mmol/g [45].

### 4.3. Preparation of the HPAM-Cr^3+^ Crosslinked System

First, stock solutions were prepared using synthetic formation water as the solvent: a HPAM solution at 10,000 mg/L and an OCR-1 solution with an effective Cr^3+^ concentration of 1000 mg/L. Subsequently, the HPAM stock solution was diluted with synthetic formation water at varying formulation ratios under constant stirring to produce HPAM solutions with target concentrations ranging from 200 to 4000 mg/L. For the crosslinking procedure, a predetermined volume of an HPAM solution was placed under stirring, and the OCR-1 stock solution was added dropwise at specified ratios. Stirring was maintained for an additional 10 min after the addition was complete. This methodology ultimately yielded crosslinked systems with varying concentrations of both HPAM and Cr^3+^.

### 4.4. Determination of Gelation Time

**Figure 10:**
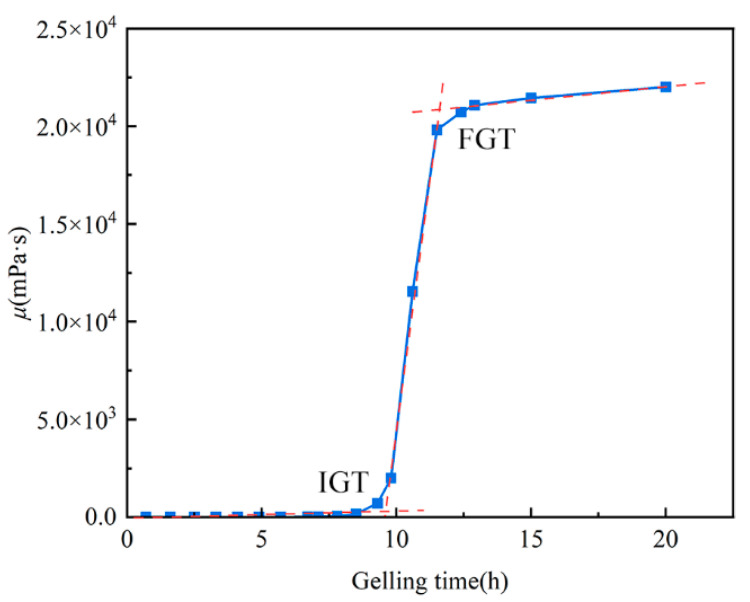
Viscosity of the Crosslinked System as a Function of Gelation Time. (Red dashed line is the trend line of the viscosity of the crosslinked system as a function of gelation time, while the crossing point of which is the Gelation Time).

The gelation time of the crosslinked system was determined using a viscosity method. The prepared HPAM-Cr^3+^ gelant was sealed and statically incubated in a constant-temperature water bath at 60 °C. The viscosity of the system at different incubation times was measured using a Brookfield viscometer (DV-II, Massachusetts, USA). During measurement, the viscometer was maintained at 60 °C with a shear rate set to 13.88 s^−1^. Figure 10 illustrates the viscosity evolution during the gelation process of the HPAM-Cr^3+^ system. The gelation process comprises two distinct stages: the Initial Gelation Time (IGT) and the Final Gelation Time (FGT) [46,47]. The IGT is defined as the point at which a marked increase in system viscosity is observed, indicating the onset of the crosslinking reaction. The FGT is identified as the time when the system viscosity reaches a stable plateau.

In this work, “stability” is defined as the ability of the formed gel to maintain its plateau storage modulus G′ over time, exhibit low static fluid-loss rate R_w_ (limited syneresis), and remain structurally continuous rather than fragmenting. This stability is not determined by composition alone; it is strongly governed by gelation time. When starting time of the gelation is aligned with the transit time of the gelant to the target position, the system forms an SSG: high, sustained G′ and low R_w_. If gelation occurs too early, rapid local over-crosslinking produces brittle, syneresis-prone OCG near the wellbore rather than a coherent bloking at depth. If gelation occurs too late, the gelant arriving in the target zone is already diluted/partially degraded by shear and retention, and only a weak or dispersed structure (SWG/CDG) is obtained, which is less stable. Thus, long-term gel stability in the reservoir is maximized when gelation time is matched to placement distance.

### 4.5. Determination of Gel Storage Modulus

Polymer gels exhibit characteristics intermediate between elastic solids and viscous liquids, embodying properties of both. The storage modulus (G′) quantifies the elastic strength of a gel. A higher storage modulus indicates greater internal frictional resistance within the gel, thereby impeding its mobility through porous media. The G′ of the HPAM-Cr^3+^ crosslinked system was measured using an Anton Paar rheometer equipped with a PP25 cone-plate rotor (diameter: 25 mm; cone angle: 1.994°; truncation gap: 102 μm). Prior to measurement, the gelant was allowed to equilibrate within the measuring cell for at least 10 min. An oscillatory frequency sweep was then performed over a range of 0.1 to 10 Hz at a fixed stress of 0.2 Pa to determine the storage modulus [45,48,49]. These G′ values serve as the key indicator of gel strength and mechanical integrity, and are used to quantitatively classify each formulation into one of the five gel states defined in Section 2.1.2.

### 4.6. Determination of Gel Static Fluid Loss Rate

First, a filter paper (diameter: 90 mm) was placed flat in a Buchner funnel and moistened with deionized water to ensure it adhered tightly to the funnel wall. A specific mass (*m*_0_) of the crosslinked gel system was then weighed and poured into the funnel, and the filtrate was collected over a period of 30 min. Subsequently, the mass of the filtrate (*m*_1_) was measured. The static fluid loss rate (R_w_) of the crosslinked system was then calculated using Equation (6).

(6) $$R_{w}=\frac{m_{1}}{m_{0}}\times 100\%$$

### 4.7. Determination of HPAM and Cr^3+^ Concentrations

#### 4.7.1. Determination of HPAM Concentration

The concentration of HPAM was determined using the starch-cadmium iodide method. Specifically, 2 mL of the test solution was pipetted into a 100 mL volumetric flask. Then, 25 mL of deionized water, 5 mL of buffer solution (pH = 5.0), and 1 mL of saturated bromine water were added sequentially. The mixture was shaken thoroughly and allowed to stand for 15 min. Subsequently, 5 mL of sodium formate solution was added, followed by shaking and a standing period of 5 min. Next, 5 mL of the starch-cadmium iodide reagent was introduced. After shaking, the mixture was left to stand for another 20 min to allow for full color development. Finally, the volumetric flask was filled to the 100 mL mark with deionized water and mixed thoroughly. The absorbance of the resulting solution was measured at 590 nm using a UV-Vis spectrophotometer.

A standard curve for HPAM concentration (ranging from 0 to 140 mg/L) in the HPAM-Cr^3+^ crosslinked system was established by following the aforementioned procedure with standard solutions of known HPAM concentrations. The absorbance of samples extracted from the sand-pack effluent was measured, and the corresponding HPAM concentration was determined from the standard curve. The original HPAM concentration in the sample was then calculated by applying the appropriate dilution factor.

#### 4.7.2. Determination of Cr^3+^ Concentration

The Cr^3+^ concentration was determined using Inductively Coupled Plasma Optical Emission Spectrometry (ICP-OES). A standard curve was first established by measuring the characteristic emission line intensity of certified chromium standard solutions with known concentrations. Subsequently, the gel samples were completely digested using aqua regia to convert all chromium species into free ions. The characteristic emission line intensity of the digested sample was then measured by ICP-OES, and the Cr^3+^ concentration was calculated based on the pre-established standard curve.

### 4.8. Propagation Experiment of the Crosslinked System in Sand-Pack

The sand-pack array consisted of 30 individual tubes (length: 200 cm, inner diameter: 1.0 cm) connected in series. Each sand-pack was packed with 60–80 mesh quartz sand using the wet-packing method. The pore volume (PV) of each sand-pack was determined by gravimetric measurement. Formation water was then injected at a constant displacement rate of 1.0 mL/min until the injection-end pressure stabilized. The equilibrium pressure was recorded, and the permeability was calculated using Darcy’s law.

The experiments evaluated propagation distance, dispersion effects, and the gel state at various locations along the sand-pack. Although the setup resembles a conventional core flood apparatus, no post-gel water flooding or oil recovery measurements were conducted in this study. Consequently, no corresponding RF–PV curves were generated. The focus was on investigating gelant transport and gelation behavior rather than evaluating recovery performance.

**Figure 11:**
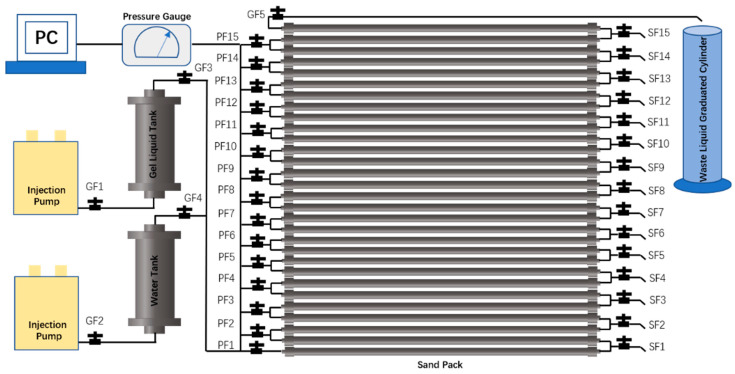
Schematic Diagram of the Displacement Process.

Experimental Procedure:

1. The sand-pack array and injection/production system were assembled inside a constant-temperature chamber according to the experimental flowchart Figure 11, with all valves initially closed. The chamber temperature was set to 60 °C. Valves GF3, PF1, GF5, and GF4 were then opened sequentially.
2. Formation water was injected at 10 mL/min for 3 PV. Subsequently, valves GF4 and GF5 were closed while valves GF1 and GF2 were opened.
3. The crosslinked system was injected at 10 mL/min for 1 PV. Valve GF3 was then closed, and a 50 mL sample was collected through valve SF1 before closing it. Additional 50 mL samples were sequentially collected from valves SF2, SF3, SF4, SF5, SF6, SF8, SF10, SF13, and the production outlet. The corresponding average distances from the injection point were 1 m, 5 m, 9 m, 13 m, 17 m, 21 m, 29 m, 41 m, 53 m, and 59 m, respectively.
4. For chemical analysis, 1 mL of each sample was diluted to 50 mL with deionized water in a volumetric flask. HPAM concentration was determined by starch-cadmium iodide spectrophotometry, while chromium ion concentration was measured using iCAP -7000 ICP-OES (Thermo Scientific, Waltham, MA, USA).
5. From each original sample, 40 mL was divided equally into two aliquots. After gelation at 60 °C, the G′ and R_w_ of the formed gels were measured separately for each aliquot.

Prior studies have indicated that porosity has negligible influence on gelation performance in high-permeability reservoirs.

## Figures and Tables

**Table 1:** Characteristic Parameters of Gels with Different Performance Categories.

Gel Morphology		Visual Appearance		G′_gel_/Pa	R_w_/%
Morphology Description	Morphology Code	Appearance Description	Appearance Code		
Stable Strong Gel	SSG	Homogeneous Transparent Viscoelastic Body	1	≥10	≤15
Stable Weak Gel	SWG	Homogeneous Transparent Viscoelastic Body	1	≥G′_HPAM_, <10	$R_{w}^{HPAM}$ ≤ R_w_ ≥ 15
Colloidal Dispersion Gel	CDG	Homogeneous Transparent Fluid	1	<G′_HPAM_	$\geq R_{w}^{HPAM}$
Unstable Gel	USG	Turbid Viscoelastic Body	1–2	≥G′_HPAM_	≥15
Over-crosslinked Gel	OCG	Heterogeneous Fluid	3	<G′_HPAM_	$\geq R_{w}^{HPAM}$

## Data Availability

The data presented in this study are available on request from the corresponding author.

## Abbreviations

The following abbreviations are used in this manuscript:

HPAM	Partially Hydrolyzed Polyacrylamide
SSG	Stable Strong Gel
SWG	Stable Weak Gel
CDG	Colloidal Dispersion Gel
USG	Unstable Gel
OCG	Over-crosslinked Gel
UGF	Un-gelled Fluid
IGT	Initial Gelation Time
FGT	Final Gelation Time
G′	Storage modulus
R_w_	Water loss rate
